# No Evolutionary Shift in the Mating System of North American *Ambrosia artemisiifolia* (Asteraceae) Following Its Introduction to China

**DOI:** 10.1371/journal.pone.0031935

**Published:** 2012-02-22

**Authors:** Xiao-Meng Li, Wan-Jin Liao, Lorne M. Wolfe, Da-Yong Zhang

**Affiliations:** 1 State Key Laboratory of Earth Surface Processes and Resource Ecology, MOE Key Laboratory for Biodiversity Science and Ecological Engineering, Beijing Normal University, Beijing, China; 2 Department of Biology, Georgia Southern University, Statesboro, Georgia, United States of America; University of Umeå, Sweden

## Abstract

The mating system plays a key role during the process of plant invasion. Contemporary evolution of uniparental reproduction (selfing or asexuality) can relieve the challenges of mate limitation in colonizing populations by providing reproductive assurance. Here we examined aspects of the genetics of colonization in *Ambrosia artemisiifolia*, a North American native that is invasive in China. This species has been found to possess a strong self-incompatibility system and have high outcrossing rates in North America and we examined whether there has been an evolutionary shift towards the dependence on selfing in the introduced range. Specifically, we estimated outcrossing rates in one native and five invasive populations and compared levels of genetic diversity between North America and China. Based on six microsatellite loci we found that, like the native North American population, all five Chinese populations possessed a completely outcrossing mating system. The estimates of paternity correlations were low, ranging from 0.028–0.122, which suggests that populations possessed ∼8–36 pollen donor parents contributing to each maternal plant in the invasive populations. High levels of genetic diversity for both native and invasive populations were found with the unbiased estimate of gene diversity ranging from 0.262–0.289 for both geographic ranges based on AFLP markers. Our results demonstrate that there has been no evolutionary shift from outcrossing to selfing during *A. artemisiifolia's* invasion of China. Furthermore, high levels of genetic variation in North America and China indicate that there has been no erosion of genetic variance due to a bottleneck during the introduction process. We suggest that the successful invasion of *A. artemisiifolia* into Asia was facilitated by repeated introductions from multiple source populations in the native range creating a diverse gene pool within Chinese populations.

## Introduction

The main challenge for populations of self-incompatible outcrossing plants is the mate acquisition phase of sexual reproduction. This part of the life cycle can be especially tenuous for invasive species because it depends on the number and distribution of genetically compatible genotypes. A biological invasion typically originates with the transport of individuals from the species' native to the introduced range. Since long-distance introduction involves a subsampling of the native range gene pool [1], [2], [3], [4] founding populations are usually initiated by small numbers of propagules that face genetic and demographic challenges that increase the probability of local extinction [5], [6].

Smaller and more isolated populations of self-incompatible wind-pollinated plants often experience increased pollen limitation [7]. Such negative effects of low abundance on individual performance and population growth known as Allee Effects can dramatically decrease rate of spread, even preventing invasion altogether [8], [9], [10]. Under such conditions, natural selection may favor the adaptive evolution of selfing [5], [11], [12], [13], [14], which will allow a rapid build-up of the population from a small number of colonists. This association between uniparental reproduction and colonizing ability is often referred to as Baker's Law [15]. Therefore, we may expect to see invasive species exhibiting breeding system transitions in the introduced range to avoid these constraints [11], [16], [17], [18], [19], [20]. By providing reproductive assurance, selfing and asexual reproduction can facilitate population establishment under conditions of low density [11], [21], [22], [23], [24]. However, efforts to explore the evolution of plant reproductive systems during biological invasion have only been made relatively recently [11], [25], [26]. Out of five case studies in which the breeding system has been estimated in both native and invasive ranges [27], [28], [29], [30], [31], only one exhibited a shift from self-incompatibility to self-compatibility following introduction to the novel range [31].

Founder events and genetic bottlenecks are generally thought to result in a reduction in the genetic diversity when compared to the total diversity present in the native range [32], [33]. Indeed, loss of genetic diversity has been reported for selectively neutral markers [34], [35], [36] and for loci under selection [37], [38]. A recent meta-analysis revealed the significant loss of both allelic richness and heterozygosity in invasive populations [39]. On the other hand, for several of successful invaders (e.g. *Ambrosia artemisiifolia*, *Bromus tectorum*, *Clidemia hirta*) the level of genetic diversity is actually greater in the introduced ranges [2], [18], [40], although the meta-analysis demonstrated that this pattern is rare [39]. One way to explain the preservation of genetic variation in the introduced range is that the colonization phase was associated with high levels of propagule pressure [16], [41], [42], thereby overcoming any Allee effects or affecting the capacity of invasive species to adapt to its new environment [43]. Under such conditions, selection for subsequent evolution of the mating system would be relaxed if reproductive success is not limited by the number of genetically compatible mates.

The goal of the present study was to document the mating system and levels of genetic variation in *Ambrosia artemisiifolia*, a plant that has invaded China. This wind-pollinated, monoecious annual is reported to possess a strong self-incompatibility mechanism and exhibit high outcrossing rates in its native North American range [44]. However, molecular studies have revealed a deficit of heterozygotes in both native and invasive populations [45], [46], [47]. This pattern may be the result of either selfing and/or biparental inbreeding. Hence, a study to quantify the degree of selfing is critical to help us understand the factors contributing to this species' Chinese invasion. We wished to determine whether the spread of this species in China was associated with an evolutionary transition from outcrossing to selfing. We had two specific objectives. First, we estimated the selfing rates from North American and Chinese populations using microsatellite markers. Second, in order to evaluate the effects of propagule pressure, we quantified the levels of genetic diversity in four native North American populations and six invasive Chinese populations using AFLP (amplified fragment length polymorphism) markers.

## Methods

### Ethics Statement

No specific permits were required for the described field studies. The sampling location is not privately-owned or protected in any way, and the field studies did not involve endangered or protected species.

### Study Species

The common ragweed, *Ambrosia artemisiifolia* L. (Asteraceae) is an aggressive annual weed of agricultural fields and disturbed sites that is native to North America and has become invasive in Europe and Eurasia [48]. *Ambrosia artemisiifolia* was introduced into China in 1930s and now occurs across a large range from Guangdong Province in the south to Heilongjiang Province in the north, and has invaded a variety of plant communities [49]. The species has a monoecious, wind-pollinated breeding system and previous work has found the mating system in its native North America to be primarily outcrossing due to a self-incompatibility system [44].

### Estimation of the Mating System

**Figure 1:**
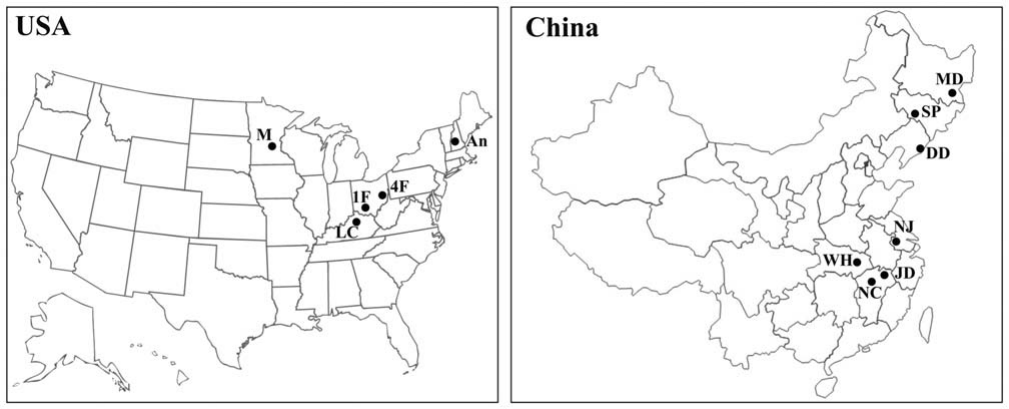
Map of *Ambrosia artemisiifolia* populations used in this study from China and North America. Population MD, SP, DD, NJ, NC, and LC were used for outcrossing rates estimation with microsatellite; MD, SP, DD, WH, NJ, NC, 1F, 4F, M, and An were for genetic diversity estimation with AFLP.

The mating system (selfing rate) was determined using microsatellite markers for one native North American population (LC) and five populations (MD, SP, DD, NJ, and NC) sampled over a large latitudinal gradient in eastern China (Figure 1). For each population, we detected the genotypes of all the seeds randomly sampled from 12 mother plants and 8 mature seeds per mother plants across six SSR loci (GenBank accession numbers: FJ595150, FJ595151, FJ595152, FJ595154, FJ595155, and FJ595156). Total genomic DNA was extracted using a plant genomic DNA extraction kit (Tiangen, China). All the SSR fragments were amplified via standard PCR in 25 µl volume containing 2.5 µl 10×PCR buffer, 2 µl 2.5 mM dNTPs, 1.5 µl 25 mM MgCl_2_, 0.5 µl 10 mM each fluorescence-labeled forward and reverse primers, 1 U Taq DNA polymerase (TaKaRa, Liaoning, China), 16.8 µl H_2_O and 1 µl (ca. 10–20 ng) genomic DNA. PCR amplifications were performed in a Bio-Rad thermal cycler as follows: an initial denaturation step at 94°C for 5 min followed by 30 cycles of 30 s at 94°C, 45 s at 50°C and 45 s at 72°C, with a final extension period of 8 min at 72°C. The fluorescence-labeled PCR products were denatured and analyzed on an ABI 3100 genetic analyzer, using the ROX 500 as an internal size standard. The genotypes were scored using GENEMAPPER software version 3.7 (Applied Biosystems).

We estimated the single-locus outcrossing rate (*t_s_*), multilocus outcrossing rate (*t_m_*), outcrossing rate between related individuals (*t_m_*−*t_s_*), inbreeding coefficient of maternal parents (*F*), and the correlation of paternity or proportion of full sibs among outcrossed progeny (*r_p_*) for all six populations using the expectation-maximization method in the MLTRWIN program (version 3.4) [50]. Standard deviations were based on 1000 bootstrap values, with options of re-sampling families. The distribution of bootstrap values was analyzed according to Friedman and Barrett [44] and Eckert and Barrett [51] to determine whether outcrossing rates differed significantly from 1.0. Plants are characterized as obligate outcrossers when outcrossing rates do not deviate from one. When values are significantly less than one the species is self-compatible and exhibits a mixed mating system.

### Analysis of Genetic Diversity

To compare genetic variation between North American and Chinese populations of *Ambrosia artemisiifolia*, leaves were collected for AFLP analysis from the six Chinese populations in addition to four from North America (Figure 1). Leaves of 11–55 individuals were sampled at random in every population, dried using silica gel, and then preserved at room temperature. Total genomic DNA from all samples was isolated using a plant genomic DNA extraction kit (Tiangen, China).

AFLP fingerprinting was performed as described by Vos et al. [52] with some minor modifications. The digestion and ligation were carried out simultaneously. In brief, 100–200 ng genome DNA was digested with 8 U *Eco*RI and 2 U *Mse*I for 3 h at 37°C and 10 min at 70°C, and simultaneously ligation adapters *Eco*RI and *Mse*I were ligated to the sticky ends of the digested DNA fragments with 80 U T4 ligase. Selective pre-amplifications were performed with primers that match the adapter sequence and contained an additional selective base at the 3′ end. After a 20-fold dilution step, the resulting products were amplified in a second round with primers containing three selective bases and fluorescently labeled with 6-FAM (Sangon, China). The selective PCR amplifications were performed with a touchdown cycling process programmed with the following temperature profile: an initial denaturation step 94°C for 2 min, followed by 13 cycles of 94°C for 30 s, 65°C for 30 s, and 72°C for 1 min with a reduction of the annealing temperature at each cycle by 0.7°C; the annealing temperature was maintained at 56°C for the remaining 23 cycles. Four pairs of primers (E-AAC/M-CAG, E-ACT/M-CAC, E-ACT/M-CTG, and E-ATG/M-CTG) were used to amplify the DNA of 254 plants from four American populations and six Chinese populations.

The selective PCR products were denatured and separated on an ABI 3100 genetic analyzer (Applied Biosystems) with a ROX 500 as internal size standard. Fragment sizes were assessed using GENEMAPPER software version 3.7 (Applied Biosystems). Allele size determinations were performed twice manually to reduce scoring errors. The multilocus AFLP profiles were scored as present or absent to create binary matrices.

Based on the observed frequencies of AFLP fragments, allelic frequencies at each locus were estimated using Bayesian method with non-uniform prior distribution proposed by Zhivotovsky [53] in AFLP-SURV 1.0 [54]. These allelic frequencies were then used as the input for the analyses of genetic diversity following the method described in Lynch and Milligan [55]. Specifically, we calculated: 1) percentage of polymorphic loci (PLP, 5% level); 2) unbiased estimates of genetic diversity (*H_j_*, analogous to *H_e_*) to estimate the within-population diversity; and the degree of genetic differentiation (*F_st_*) among populations within each of the continents.

## Results

### Mating System

Both Chinese and North American *A. artemisiifolia* populations displayed high outcrossing rates (Table 1). The multilocus outcrossing rates (mean ± S.D.) estimated from microsatellites for Chinese populations ranged from 0.928±0.042 to 1.000±0.000. These values are not significantly different from 1.0, indicating a completely outcrossing mating system for Chinese ragweed populations. The native North American population was also completely outcrossing, with a multilocus outcrossing rate of 1.000±0.000 (Table 1).

**Table 1:** Estimates of mating system parameters for the six populations of *Ambrosia artemisiifolia* based on six microsatellite loci.

Population	*t_m_*	*t_s_*	*t_m_−t_s_*	*F*	*r_p_*
**North America**					
LC	1.000 (0.000)	0.937 (0.020)	0.063 (0.020)	0.008 (0.020)	0.018 (0.012)
**China**					
MD	0.979 (0.018)	0.913 (0.033)	0.065 (0.028)	0.002 (0.009)	0.034 (0.016)
SP	0.942 (0.036)	0.763 (0.040)	0.179 (0.025)	0.000 (0.000)	0.122 (0.047)
DD	0.928 (0.042)	0.753 (0.051)	0.175 (0.035)	0.000 (0.000)	0.081 (0.037)
NJ	1.000 (0.000)	0.923 (0.024)	0.077 (0.024)	0.000 (0.000)	0.028 (0.017)
NC	0.982 (0.013)	0.865 (0.032)	0.118 (0.027)	0.001 (0.009)	0.070 (0.032)

The values in brackets are S.D. The *t_m_*, multilocus outcrossing rate; *t_s_*, single-locus outcrossing rate; *t_m_−t_s_*, outcrossing rate between related individuals; *F*, inbreeding coefficient of maternal parents; *r_p_*, the correlation of paternity or proportion of full sibs among outcrossed progeny.

The estimates for the outcrossing rate between related individuals (*t_m_*−*t_s_*) were low for Chinese populations (range: 0.065±0.028 to 0.179±0.025) and North American population (0.063±0.020). These values were significantly greater than zero, suggesting some degree of biparental inbreeding in both Chinese and North American *A. artemisiifolia* populations. The maternal inbreeding coefficients (*F*) were less than 0.008±0.020 and did not differ from zero for all populations, indicating obligate outcrossing. The correlations of paternity (*r_p_*) were 0.028–0.122 in Chinese populations, suggesting that the populations consisted of a large number of pollen donor parents. However, there was a higher number of pollen donor parents in the North American population (Table 1).

### Genetic Diversity and Structure

The four pairs of FAM-*Eco*RI/*Mse*I primer combinations in the ten populations yielded a total of 322 AFLP fragments, of which 321 (99.7%) were polymorphic at the 5% level. North American and Chinese populations shared 320 out of the 322 bands. Only two bands were private between continents: one band occurred in four North American populations but was absent in China while another occurred in three Chinese populations but was not found in North American populations.

Overall genetic diversity was similar in North America and China (Table 2). The proportion of polymorphic loci for North American populations and Chinese populations was 82.2% and 78.7% respectively. The unbiased estimation of gene diversity ranged from 0.262–0.289 for the four North American populations and from 0.263–0.283 for six Chinese populations. The *F_st_* values indicate that there is a greater degree of genetic structure in the introduced Chinese range compared to North America. Approximately 94% of the genetic variation exists within North American populations compared to 87% in the invasive Chinese range (Table 2).

**Table 2:** Genetic diversity indices for Chinese and North American populations of *Ambrosia artemisiifolia* based on AFLP markers.

Population	N	PLP (%)	*H_j_*	*F_st_*
**North America**				
1F	11	81.1	0.280 (0.009)	
4F	11	80.4	0.262 (0.010)	
M	55	87.3	0.276 (0.008)	
An	15	80.1	0.289 (0.009)	
**Total North America**			0.295	0.060
**China**				
MD	28	80.4	0.280 (0.009)	
SP	26	78.3	0.283 (0.009)	
DD	20	75.8	0.263 (0.009)	
WH	29	79.2	0.272 (0.009)	
NJ	30	80.7	0.269 (0.009)	
NC	29	77.6	0.265 (0.009)	
**Total China**			0.311	0.127

N is the number of scored individuals; PLP is the proportion of polymorphic loci at the 5% level; *H_j_* is Nei's genetic diversity; *F_st_* is Wright's *F_st_*.

## Discussion

The plant mating system is subject to environmental and genetic influences that operate at both ecological and evolutionary time scales [56]. Limited mating often reduces the seed production of individuals in outcrossing populations [57], and the ability to self-fertilize can overcome this constraint on reproductive success [58], [59]. Invasions often begin with a small number of founders and subsequently there is the potential for compatible mates to be a limiting resource. Under these conditions, natural selection would be expected to favor uniparental reproduction in colonizing populations [11]. So far, only one study in the literature clearly discovered the shift from self-incompatibility to self-compatibility in the invasive range [31].Our study provides the first detailed evidence of the mating system of *A. artemisiifolia* in its invasive Asian range. The main result is that the outcrossing rates are very high and do not deviate from one in both native North American and invasive Chinese populations. In addition the indirect estimates of the paternity correlations (*r_p_*) were low suggesting that all of the populations possessed a high number of pollen donor parents contributing to each maternal plant. Moreover, the maternal inbreeding coefficients (*F*) were nearly equal to zero for all the populations, indicating the absence of inbreeding. Combined with the uniformly high outcrossing rates in North America reported by Friedman and Barrett [44], these results indicate that there has not been an evolutionary transition from the fully outcrossing sexual system that exists in North America during *A. artemisiifolia*'s Asian invasion. Moreover, our results suggest that the positive fixation indices *F*_IS_ reported in European populations of *A. artemisiifolia* [45], [46], [47] have likely not resulted from selfing, but from other factors such as the Wahlund effect, which refers to the reduction of heterozygosity due to subpopulation structure.

Since colonizing populations often establish with only a subset of the genotypes present in the native range, we expect less genetic variation to exist in introduced populations when compared to those in the native range [3]. However, AFLP markers revealed similarly high levels of polymorphism and genetic diversity in both Chinese and North American populations, although there was more structure present in Chinese populations. *Ambrosia artemisiifolia* has also invaded Europe where populations contain similar levels of allelic diversity and heterozygosity as in North America [45], [46], [47]. Perhaps the most parsimonious way to explain this departure from theoretical expectations is that the colonization phase experienced high levels of propagule pressure. If these initial populations were founded by repeated introductions from multiple native source populations, subsequent gene flow and admixture among historically divergent populations in the native range would result in high levels of genetic variation [16], [60], [61].

One of the goals of research on biological invasions is to understand the ecological and genetic factors that contribute to successful establishment and subsequent geographic spread. It is generally believed that owing to being limited to a single bout of reproduction, annual species should be primarily selfing and not possess a self-incompatibility system [2]. This association would be expected to be especially true for colonizing populations of invasive species owing to the dangers of reproductive failure resulting from low population densities that can occur along an invasion front [62]. Following this reasoning, it was assumed that *A. artemisiifolia* would be a selfer throughout its invasive range in Europe and Asia. However, it is apparent that the species has a highly outcrossed mating system in both its native North American range [44] and in Asian populations in China. Clearly this species has avoided the negative Allee Effects resulting from low abundance and self-incompatibility. The broad geographic distribution of ragweed indicates that while it is an outcrosser, the species exhibits some classic features of a successful invading colonizer [15] by being a rapid growing annual that produces prolific quantities of seeds [44]. Furthermore, not only are invasive populations outcrossing, but high levels of genetic variation are generated during reproduction from multiple paternity arising from the combination of copious quantities of pollen along with flowers that contain only a single ovule.
